# Patient education needs in severe asthma, a pilot study

**DOI:** 10.1186/s12890-024-02960-8

**Published:** 2024-03-15

**Authors:** Rodolphe Laurence, Julien Ancel, Maëva A. Devilliers, Sophie Carre, Sandra Dury, Valérian Dormoy, Gaëtan Deslée, Jeanne-Marie Perotin

**Affiliations:** 1 Department of Respiratory Diseases, University Hospital of Reims, Reims, France; 2 University of Reims Champagne-Ardenne (URCA), Inserm UMR-S 1250, SFR Cap-Santé, Reims, France; 3 Transversal Unit of Patient Education (UTEP), University Hospital of Reims, Reims, France; 4 CRISALIS/F-CRIN INSERM Network, Service des Maladies Respiratoires, CHU Maison Blanche, Inserm UMR-S, 45 rue Cognacq-Jay, 1250, 51092 Reims cedex, France

**Keywords:** Severe asthma, Phenotype, Patient education, Eosinophils, Age

## Abstract

**Background:**

Severe asthma is characterized by frequent exacerbations, altered lung function, and impaired quality of life. Tailored patient education allows for the improvement of both asthma management and quality of life. Our study aimed to assess the needs of severe asthma patient in therapeutic education, according to previous therapeutic patient education background and asthma phenotype.

**Methods:**

Consecutive patients monitored for severe asthma in a tertiary referral center were considered for inclusion and answered a questionnaire detailing their patient education needs and the topics they would like to discuss. Asthma history, clinical and biological data, and lung function results were recorded.

**Results:**

Fifty-three patients were included and 47 (88.7%) expressed at least one need. The most frequently selected topics were “life with asthma” (83%), “treatment use” (68%), and “exacerbation management” (60%), independent of previous participation in a patient education program dedicated to asthma. Patients of older age at inclusion, uncontrolled asthma, and T2-high phenotypes were associated with different profiles of patient education needs.

**Conclusion:**

Our study identified frequent and various patient educational needs among severe asthmatics, highlighting the importance of an in-depth assessment of severe asthmatics expectations and the crucial need for the development of dedicated educational tools.

**Supplementary Information:**

The online version contains supplementary material available at 10.1186/s12890-024-02960-8.

## Background

Severe asthma affects 3–10% of an estimated 300 million asthma sufferers worldwide. Severe asthma is defined by the requirement for treatment with high-dose inhaled corticosteroids and a co-controller or systemic corticosteroids for ≥ 50% of the previous year to prevent it from becoming uncontrolled or remaining uncontrolled despite this therapy [1]. As a major cause of morbidity, asthma is an important public health issue given its impact on work productivity and the costs associated with disease management and healthcare resource use [2]. A better understanding of the mechanisms involved in severe asthma, together with the onset of biologics, has led to an improvement in the management of patients. However, asthma remains uncontrolled for many patients, which is associated with a poor quality of life, as well as social, professional, and health burdens [3].

Patient education aims to reinforce therapeutic alliance, adherence to medical care, and patient skills including self-management, to improve asthma management and control, as well as quality of life [4–6]. Patient education programs are thought to be tailored for each patient depending on their needs and usually include information about asthma, training for the correct use of inhaler devices, training in guided self-management of asthma symptoms relying on symptoms and peak expiratory flow self-evaluation and the use of written action plan [7]. The needs of severe asthma patients in terms of patient education might differ from mild to moderate asthmatics and therefore require a dedicated therapeutic patient education (TPE) program. A recent expert consensus identified 16 patient-reported outcomes (PROs) in severe asthma, including asthma symptoms, comorbiditiy-related symptoms and quality of life -related outcomes [8].

The PENSA study (Patient Education Needs in Severe Asthma, MR-004 29,062,021) aimed to assess self-reported needs in therapeutic education of severe asthma patients, according to previous patient education background, baseline characteristics, and asthma phenotype.

## Methods

### Study design

The PENSA study is a pilot single-center cross-sectional study performed from July 2021 to June 2022, recruiting severe asthma patients from the Severe Asthma clinics, Department of Respiratory Diseases, University Hospital of Reims, France.

Consecutive adult patients with a diagnosis of severe asthma (ATS-ERS definition) [1] were asked to participate in the study, regardless of smoking history or comorbidities. Patients with evidence of other chronic pulmonary disease, including chronic obstructive pulmonary disease (COPD), were excluded.

The study was approved by the Reims University Hospital. It was conducted under the methodology MR-004 of the French data protection authority (Commission Nationale de l’Informatique et des Libertés, CNIL; 29,062,021). All patients provided written informed consent.

TPE program, when provided before the enrolment of the patients, consisted in one or several individual sessions, whose content was personalized depending on the patients’ expressed needs, and included at least information about asthma, training for the correct use of inhaler devices, training in guided self-management of asthma symptoms relying on symptoms and peak expiratory flow self-evaluation and the use of written action plan, as recommended [8–10].

The primary objective was to describe severe asthma patients’ self-determined needs in terms of patient education. The secondary objective was to compare severe asthma patient education needs depending on previous participation in patient education programs dedicated to asthma and on clinical and biological phenotypic characteristics (age at inclusion, age at asthma onset, time from severe asthma diagnosis, BMI, atopy, ACT score, frequent severe exacerbations, FEV_1_, blood eosinophil counts).

### Collected data

At inclusion, the patient data was collected from medical files, including demography, smoking history, asthma history, exacerbations, comorbidities including obesity (BMI > 30 kg/m²), and treatment including long-term oral corticosteroids and biologics treatment (1st line, 2nd line). Ex-smoking was defined as smoking cessation for at least 12 months. Severe exacerbations were defined as requiring systemic corticosteroids for more than 48 h, emergency department visit or hospitalisation. Asthma control was assessed at inclusion using the Asthma Control Test (ACT) score. Treatment adherence was assessed using the Medication Adherence Report Scale (MARS). The results of respiratory functional tests (FEV_1_, FEV_1_/FVC) and the highest blood eosinophil counts (before biologic treatment when used) were recorded, as well as previous participation in a TPE program. Fixed airway obstruction was defined as FEV_1_/FVC < 70% after bronchodilation. Patients’ occupation was classified into 5 groups: managers and intellectual professions, technical professions, services, unemployed, and students. Older age, high blood eosinophil count and uncontrolled asthma (low ACT score, ≥ 2 severe acute exacerbation) and/or reduced FEV_1_ were analysed as phenotypic traits.

Patients were asked to complete an original questionnaire listing a series of 20 topics in 5 domains that they wished to discuss with healthcare providers (Suppl Table 1). These topics encompassed security needs including “red flags” (i.e. signs that must alert the patient on a loss of asthma control or on the severity of an exacerbation), knowledge about their pathology, living with asthma, and sharing with fellow patients. Additionally, space was provided for patients to include any other topic they deemed relevant.

### Statistical analyses

Descriptive data were expressed as numbers (percentages) and means ± SD or medians [25th -75th percentiles], depending on the distribution. Comparisons of quantitative variables between groups were performed using Mann-Whitney U tests. Comparisons for qualitative variables were performed using Pearson’s chi-squared test or Fisher’s exact tests according to distribution. P-value < 0.05 was considered significant.

## Results

### Patients’ characteristics

Fifty-three patients were included, mean age of 54, 56% women, 34% obese, with a median time from severe asthma diagnosis of 3 [1-6.5] years (Table 1). Despite good treatment adherence in more than 90% of the patients (defined as a total MARS score ≥ 21), asthma was frequently uncontrolled: 58.5% had an ACT score < 20, and 47.2% had ≥ 2 severe exacerbations requiring oral corticosteroids in the last year. A fixed airflow obstruction was observed in 54.7% of the patients. At inclusion, 30.2% received oral corticosteroids and 92% biologics.

**Table 1:** Patients’ characteristics

Number of patients	53
Age (yrs) (mean ± SD)	53.5 ± 13.6
Age at severe asthma diagnosis (yrs) (mean ± SD)	49.7 ± 15.1
Female gender (n, %)	30 (56.6)
Body mass index (kg/m²) (mean ± SD)	28.9 ± 5.9
≥ 30 (n, %)	18 (33.9)
Smoking history	
Current smoker (n, %)	2 (3.8)
Ex-smoker (n, %)	24 (45.3)
Pack-year (*n* = 26) (mean ± SD)	15.7 ± 13.5
Comorbidities	
Nasal polyps (n, %)	23 (43.4)
GERD (n, %)	20 (37.7)
Allergic rhinitis (n, %)	12 (22.6)
Occupation category	
Manager and intellectual professions (n, %)	8 (15.1)
Technical professions (n, %)	20 (37.7)
Services (n, %)	17 (32.1)
Unemployed (n, %)	6 (11.3)
Students (n, %)	2 (3.8)
Treatment adherence (MARS score ≥ 21)	48 (90.6)
ACT score (mean ± SD)	17.0 ± 5.3
< 15 (n, %)	16 (30.2)
15–19 (n, %)	15 (28.3)
≥ 20 (n, %)	22 (41.5)
Exacerbations	
In the last year (n, %)	35 (66)
Number per patient (median[25th -75th ]	1 [0–4]
≥ 2 severe exacerbations in the last year (n,%)	25 (47.2)
Blood eosinophils (10^9^/L) * (median[25th -75th ]	0.60 [0.4–1.0]
< 150 (n, %)	2 (3.8)
150–299 (n, %)	2 (3.8)
≥ 300 (n, %)	49 (92.4)
Aeroallergen sensitization (n, %)	26 (49.1)
FEV_1_ post bronchodilation, % (mean ± SD)	80.2 ± 18.6
FEV_1_/FVC (mean ± SD)	68.2 ± 9.9
Treatment at inclusion	
Inhaled corticosteroid (n, %)	53 (100)
Long-acting beta2 agonist (n, %)	53 (100)
Long-acting muscarinic antagonist (n, %)	13 (24.5)
Montelukast (n, %)	27 (50.9)
Long-term oral corticosteroid (previous or current) (n, %)	16 (30.2)
OCS dose (mg/day) (mean ± SD)	12.7 ± 7.7
Biologic therapy (current) (n, %)	49 (92.5)
1st line (n, %)	25 (51.0)
≥2nd line (n, %)	24 (49.0)
Previous TPE program (n, %)	21 (39.6)
Time from the last TPE program > 1 year (mean ± SD)	11 (20.8)

Data are expressed as number (percentage), mean ± SD or median [25th -75th ]

*: highest count before biologic treatment

GERD: Gastro-eosophageal reflux disease; MARS: Medical Adherence Report Scale; ACT: Asthma Control Test; TPE: Therapeutic patient education

### The needs of severe asthma patients in therapeutic patient education

Forty-seven patients (88.7%) identified at least one topic, with a mean of 9 ± 5 topics per patient. Six patients (11.3%) did not select any topic. When compared with other patients, those 6 patients were significantly younger (37.3 ± 18.3 vs. 55.5 ± 11.6 years old, *p* = 0.001).

The most frequent topics selected by the patients (Table 2) were related to security needs, including treatment use (*n* = 36, 68%, including corticosteroids, *n* = 20, 37.7%, and/or biologics, *n* = 23, 43%), and exacerbation management (*n* = 32, 60%). The patients were also interested in acquiring knowledge about severe asthma as a disease (*n* = 28, 53%) and frequent comorbidities (*n* = 26, 49%).

**Table 2:** Selected topics

Topic requested	Total
Number of patients	53
No topic selected	6 (11.3)
Security needs	40 (75.5)
Treatment use	36 (67.9)
Inhaled treatment	13 (24.5)
Corticosteroids	20 (37.7)
Biologics	23 (43.4)
Symptoms and exacerbation management	32 (60.4)
Asthma Control	20 (37.7)
Red flags	14 (26.4)
Pathology’s knowledge	36 (67.9)
What is severe asthma?	28 (52.8)
Asthma symptoms	11 (20.8)
Living with asthma	44 (83.0)
Comorbidities	26 (49.1)
Sports	25 (47.2)
Nutrition/diet	19 (35.9)
Pollutants	15 (28.3)
Emotion	14 (26.4)
Allergies	12 (22.6)
Social rights	10 (18.9)
Family	7 (13.2)
Sharing with fellow patients	18 (34.0)
Group therapy	14 (26.4)
Partner patient	5 (9.4)

Data are expressed as number (percentage)

Forty-four patients (83%) expressed an interest in “life with asthma” topics, including symptoms-triggering factors such as allergies (*n* = 12, 23%) and pollutants (*n* = 15, 28%), sports (*n* = 25, 47%), nutrition (*n* = 19, 36%) or emotions (*n* = 14, 26%). One-third of patients (*n* = 18, 34%) would like to share with fellow patients.

### The needs of severe asthma patients depending on patients features

**Fig. 1:**
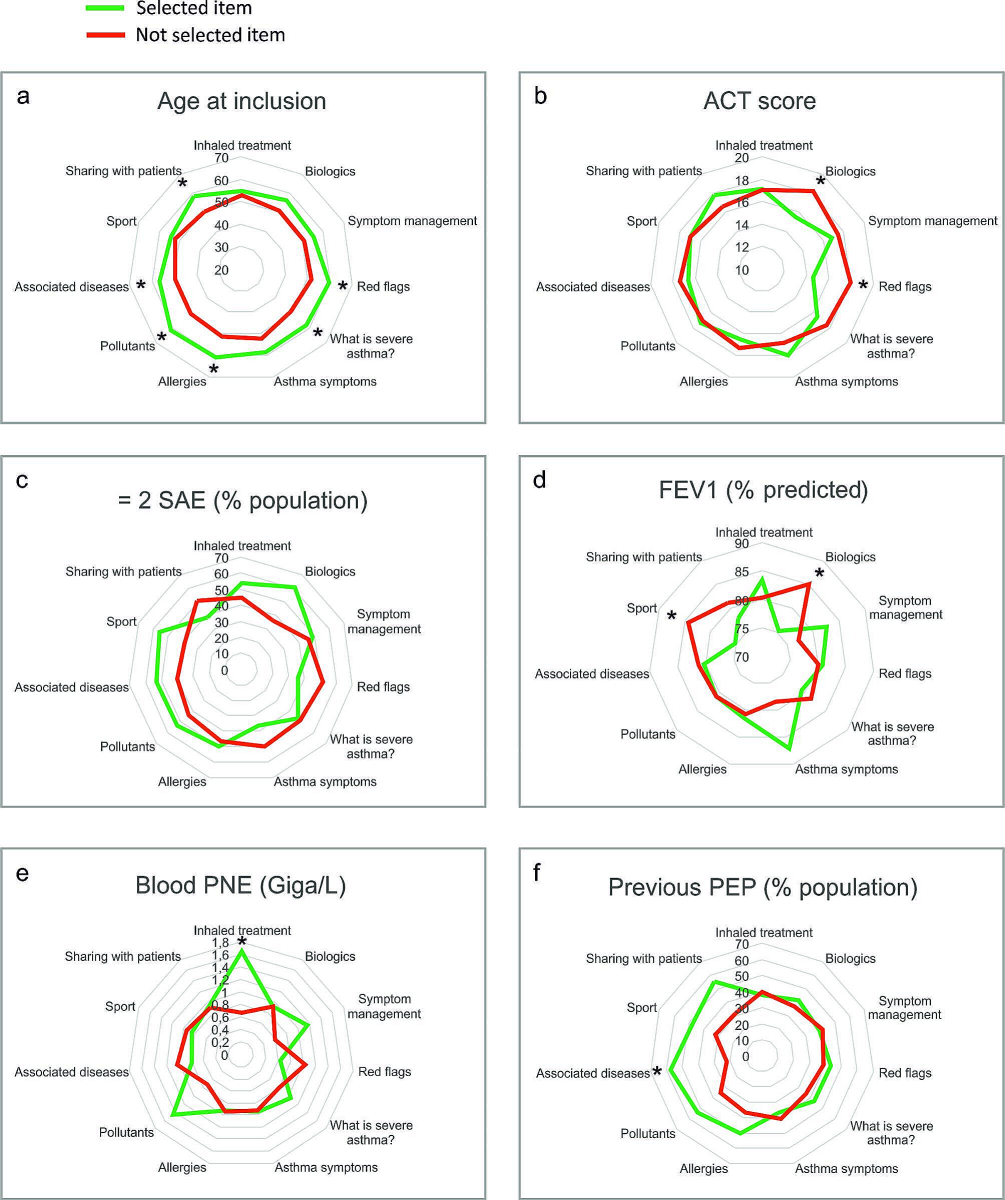
Phenotypic characteristics in patients who did select an item (green) and those who did not select the same item (red) among 11 educational needs.: (**a**) mean age at inclusion, (**b**) mean ACT score, (**c**) percentage of patients with ≥ 2 severe asthma exacerbations in the last year, (**d**) mean FEV_1_ percentage of predicted value, (**e**) mean blood eosinophils value and (**f**) percentage of patients who previously benefit from TPE program. The scales for mean values or percentage are indicated in the center of the circles, corresponding to the values for each light grey concentric circle. *: *p* < 0.05

We performed an exploratory analysis of patients’ expectations depending on clinical and biological phenotypic characteristics, and previous participation in a TPE program dedicated to asthma. We observed different profiles of patients in terms of expressed needs (Fig. 1, Suppl Table 2).

Older age at inclusion was associated with a higher number of expressed needs (*p* = 0.001) in a broad range of topics including knowledge about severe asthma (*p* = 0.005), red flags (*p* = 0.027), triggering factors (allergies *p* = 0.016, pollutants *p* = 0.003), and comorbidities (*p* = 0.038). Older patients were also more frequently interested in sharing with fellow patients (*p* = 0.019) when compared to younger individuals.

Patients with uncontrolled asthma (either low ACT score, ≥ 2 severe acute exacerbation (SAE)) or reduced FEV_1_, were interested in items regarding biologic treatments, and red flags. In addition, the patients with a lower FEV_1_ value more frequently selected the “sports” item (*p* = 0.040).

Higher eosinophil blood count was associated with needs regarding inhaled treatment, exacerbation management, and “pollutants” topics (*p* = 0.013, *p* = 0.052, and *p* = 0.055 respectively).

Age at diagnosis, time from severe asthma diagnosis, atopy, BMI or occupation category were not associated with clinically relevant selected topics.

Twenty-one patients (39.6%) benefited from previous TPE program in asthma, including 10 (18.9%) in the last year. Baselines characteristics did not differ between patients who had participated in the TPE program end those who had not, except for ACT score distribution (Suppl Table 3). We did not find any differences in terms of patient education needs between patients who previously participated in a TPE program and those who did not, except for the topic “associated disease”. Amongst patients with previous TPE, those with older participation (> 1 year ago) tended to be more frequently interested in red flags (*p* = 0.055) and allergies (*p* = 0.055) topics.

## Discussion

Our pilot study identified that severe asthmatics expressed frequent and various educational needs. We also observed, for the first time to our knowledge, that several patients’ phenotypes are associated with different profiles of patient educational needs.

Patient education plays a critical role in the management of asthma, as in many other chronic diseases. However, the availability of asthma education programs at a national scale, and regional disparities between asthma prevalence and asthma education programs location limit the number of patient that can benefit from asthma TPE [9, 10]. Usual asthma TPE program includes self-management education, asthma information, and skills training as key components, adjusted and adapted for both sociocultural context and patient background and expectations [7]. Other approaches, like mindfulness interventions, may have a long-term impact on psychological symptoms in asthma patients [11]. Recently, there has been an emphasis placed on divergent perspectives and priorities between patients and healthcare providers. Healthcare providers often focus more on the physical aspects of the disease [12] while patients expect to enhance their quality of life and seek shared decision-making discussions between doctors and themselves [13]. A recent expert consensus proposed 6 patient-reported outcome measures (PROMs), to be included into severe asthma routine care including assessment of control, dyspnea, quality of life and drug dispensation and adherence [8]. In our study, 89% of the severe asthmatics expressed at least one educational need, regardless of whether they previously participated in TPE program or not. “Security needs”, such as treatments and side effects, identification of alert red flags, and exacerbation management were selected by 75% of the patients. These results confirm that the so-called “security needs” should be systematically part of TPE programs provided to severe asthmatics. They also suggest that severe asthmatics’ education needs are not completely fulfilled by the participation in a standard TPE program as described above, suggesting the interest in repeating educational sessions to maintain the long-term efficacy of education interventions [5, 8], especially regarding security needs. Results also suggest a possible suboptimal communication between healthcare providers and patients, reflecting the need for a standardized assessment of patients’ needs, and a larger range of educational tools. These results highlight the importance of considering individualized treatment plans and incorporating a multidisciplinary approach within healthcare teams.

Our study identified that different educational needs were expressed by three main phenotypes of severe asthma: uncontrolled asthma and/or reduced FEV_1_, higher blood eosinophil count, and older age. In our study, uncontrolled asthma was frequent, representing more than 50% of the population. French and European severe asthma registries previously reported similar to higher rates of uncontrolled severe asthma, ranging from 54 to 100% [14, 15]. As expected, severe asthmatics with uncontrolled asthmaand/or reduced FEV_1_ were interested in topics related to control improvement and severe exacerbation management, including biologics treatments and identification of red flags. Patients with high blood eosinophils count, which is a known risk factor for frequent and severe exacerbations, were interested in symptoms and exacerbation management. In addition, we observed that patients with lower FEV_1_ values were interested in the “sports” topic. Previous studies identified decreased physical activity in asthmatic patients when compared to control groups [16]. Recent studies showed that pulmonary rehabilitation [17, 18] might benefit patients with severe asthma on health-related quality of life and anxiety and depression symptoms.

Previous studies have shown that asthmatic patients of older age are characterized by worse airway obstruction, higher levels of non-T2 inflammatory cytokines (IFN-gamma, IL-17 A, IL-8) in induced sputum, and a reduced response to treatment [19]. In our study, older age at inclusion (but not the age of asthma onset nor the time from severe asthma diagnosis) was associated with a higher number of expressed needs across a broad range of educational topics. No previous study focusing on asthma education for older patients has been conducted previously. A recent Canadian study suggested that a high number of patients aged 60 or older would be interested in using mobile health for health information or discussing with healthcare team members [20], indicating that educational tools using electronic devices or video calls could be developed and recommended to severe asthmatics of older age.

Our study does have several limitations. Its design as a pilot cross-sectional study conducted at a single tertiary referral center, the limited number of patients included, the over-representation of T2-high phenotype, and the frequent treatment using biologics should be taken into consideration and may limit the generalizability of the results. Despite these limitations, it is worth noting that the population under investigation was well characterized, with a prospective recruitment approach. To our knowledge, our study was the first to focus on the educational needs of severe asthmatics. Further research on a larger scale, involving multiple centers and/or at a national level, would be valuable to further develop and expand upon these findings.

## Conclusions

Our study confirmed frequent and various patient educational needs amongst severe asthmatics, and suggested different profiles of needs in three clinical-biological phenotypes. If confirmed in large, national-scale studies, those results would highlight the need for an in-depth and standardised assessment of the severe asthmatics’ expectations and needs regarding therapeutic patient education, and for the development of dedicated educational tools.

### Electronic supplementary material

Below is the link to the electronic supplementary material.

Supplementary Material 1

Supplementary Material 2

Supplementary Material 3

Supplementary Material 4

## Data Availability

The data that support the findings of this study are available from the corresponding author upon reasonable request.
