# Supplementary material for: Patient education needs in severe asthma, a pilot study
Source: BMC Pulm Med. 2024 Mar 15;24:134. doi: 10.1186/s12890-024-02960-8 (PMC10943831; doi:10.1186/s12890-024-02960-8)
Supplement: Supplementary file 2 — Supplementary Material 2 [file 12890_2024_2960_MOESM2_ESM.docx]

Supplementary Table 2. Educational needs depending on patient’s clinical and biological phenotypes

|  |  | **Security needs** | | | | **Knowledge about asthma** | | **Living with asthma** | | | | **Sharing with fellow patients** |
| --- | --- | --- | --- | --- | --- | --- | --- | --- | --- | --- | --- | --- |
|  |  | Inhaled treatment | Biologics | Symptoms and exacerbation management | Red flags | What is severe asthma? | Asthma symptoms | Allergies | Pollutants | Associated diseases | Sports |  |
| **Age at inclusion** | |  |  |  |  |  |  |  |  |  |  |  |
|  | p value | 0.333 | 0.062 | 0.114 | **0.027** | **0.005** | 0.085 | **0.016** | **0.003** | **0.038** | 0.300 | **0.019** |
|  | selected need | 54.8±11.8 | 56.7±10.8 | 55.4±12.1 | 59.5 ± 13.5 | 57.9 ± 11.1 | 58.5±13.8 | 60.8 ± 8.9 | 61.5 ± 10.6 | 56.8 ± 11.1 | 54.5±12.5 | 58.9 ± 13.4 |
|  | not selected need | 52.9±14.4 | 50.8±15.3 | 50.8±15.3 | 51.4 ± 13.5 | 48.6 ± 14.6 | 52.2±13.4 | 51.2 ± 14.2 | 50.1 ± 13.6 | 50.0 ± 15.4 | 52.6±14.7 | 50.7 ± 13.0 |
| **ACT score** | |  |  |  |  |  |  |  |  |  |  |  |
|  | p value | 0.476 | **0.027** | 0.336 | **0.024** | 0.220 | 0.252 | 0.337 | 0.470 | 0.323 | 0.481 | 0.189 |
|  | selected need | 17.2±5.6 | 15.5 ± 4.4 | 16.8±5.7 | 14.6 ± 5.9 | 16.5±5.6 | 18.0±6.2 | 16.5±5.4 | 17.3±4.8 | 16.7±5.2 | 17.0±5.7 | 17.9±5.1 |
|  | not selected need | 17.1±5.3 | 18.3 ± 5.8 | 17.4±4.6 | 17.9 ± 4.9 | 17.6±4.9 | 16.8±5.1 | 17.3±5.4 | 17.0±5.6 | 17.4±5.6 | 17.1±5.0 | 16.6±5.4 |
| **≥ 2 SAE** | |  |  |  |  |  |  |  |  |  |  |  |
|  | p value | 0.751 | **0.070** | 0.528 | 0.246 | 0.564 | 0.322 | 0.540 | 0.397 | 0.248 | 0.173 | 0.283 |
|  | selected need | 7/13 | 14/23 | 15/31 | 5/14 | 13/28 | 4/11 | 6/12 | 8/15 | 14/26 | 14/11 | 7/18 |
|  | not selected need | 18/40 | 11/30 | 10/22 | 20/39 | 12/25 | 21/42 | 19/41 | 17/39 | 11/27 | 11/28 | 18/35 |
| **FEV_1_** | |  |  |  |  |  |  |  |  |  |  |  |
|  | p value | 0.294 | **0.025** | 0.301 | 0.899 | 0.662 | **0.084** | 0.913 | 0.986 | 0.837 | **0.040** | 0.0.536 |
|  | selected need | 83.5±18.1 | 75.4 ± 17 | 82.4±18.2 | 80.7±14.9 | 79.1±20.8 | 87.1±10.9 | 81.4±16.9 | 80.8±23.9 | 80.4±18.4 | 75.4 ± 16.1 | 77.9±14.6 |
|  | not selected need | 80.3±18.3 | 85.2 ± 17.9 | 77.0±19.2 | 80.0±19.9 | 81.4±16.3 | 78.4±19.9 | 80.7±18.6 | 80.9±15.4 | 81.4±18.0 | 84.4 ± 19.9 | 81.3±20.5 |
| **Blood PNE, G/L** | |  |  |  |  |  |  |  |  |  |  |  |
|  | p value | **0.013** | 0.987 | **0.052** | 0.298 | 0.489 | 0.974 | 0.977 | **0.055** | 0.491 | 0.769 | 0.949 |
|  | selected need | 1.66±2.26 | 0.93±1.57 | 1.15 ± 1.52 | 0.63±0.39 | 1.03±1.61 | 0.93 ± 0.60 | 0.91±0.63 | 1.44±2.14 | 0.80±0.62 | 0.87±0.58 | 0.94±0.64 |
|  | not selected need | 0.68±0.47 | 0.92±0.66 | 0.59 ± 0.50 | 1.03±1.41 | 0.80±0.60 | 0.92 ± 1.36 | 0.93±1.38 | 0.71±0.51 | 1.04±1.66 | 0.97±1.62 | 0.91±1.45 |
| **Previous TPE program** | |  |  |  |  |  |  |  |  |  |  |  |
|  | p value | 0.594 | 0.412 | 0.548 | 0.508 | 0.410 | 0.545 | 0.306 | 0.166 | **0.009** | 0.185 | **0.081** |
|  | selected need | 5/13 | 10/23 | 12/31 | 6/14 | 12/28 | 4/11 | 6/12 | 8/15 | 15/26 | 12/25 | 10/18 |
|  | not selected need | 16/40 | 11/30 | 9/22 | 15/39 | 9/25 | 17/42 | 15/41 | 13/38 | 6/27 | 9/28 | 11/35 |
| ACT: Asthma Control Test; PNE: Polynuclear eosinophil; SAE: severe asthma exacerbation in the last year; TPE: therapeutic patient education | | | | | | | | | | | | |
