# Supplementary material for: Patient education needs in severe asthma, a pilot study
Source: BMC Pulm Med. 2024 Mar 15;24:134. doi: 10.1186/s12890-024-02960-8 (PMC10943831; doi:10.1186/s12890-024-02960-8)
Supplement: Supplementary file 3 — Supplementary Material 3 [file 12890_2024_2960_MOESM3_ESM.docx]

Suppl Table 1. Questionnaire used to assess self-reported needs in therapeutic education of severe asthma patients, in English and in French languages.

| Please select all the topics that you wish to discuss with the healthcare team | | | |
| --- | --- | --- | --- |
|  |  |  |  |
| ⬜ | Knowledge about asthma | | |
|  |  | ⬜ | What is sever asthma? |
|  |  | ⬜ | Asthma symptoms |
|  |  | ⬜ | Asthma fakes |
| ⬜ | Treatment use | | |
|  |  | ⬜ | Inhaled treatment |
|  |  | ⬜ | Oral corticosteroids |
|  |  | ⬜ | Biologics |
| ⬜ | Control and symptoms | | |
|  |  | ⬜ | Red flags |
|  |  | ⬜ | Symptoms and exacerbation management |
|  |  | ⬜ | Asthma control |
| ⬜ | Living with asthma | | |
|  |  | ⬜ | Allergies |
|  |  | ⬜ | Pollutants |
|  |  | ⬜ | Associated diseases |
|  |  | ⬜ | Sports |
|  |  | ⬜ | Emotions |
|  |  | ⬜ | Pregnancy |
|  |  | ⬜ | Family |
|  |  | ⬜ | Social rights |
|  |  | ⬜ | Nutrition/Diet |
| ⬜ | Sharing with fellow patients | | |
|  |  | ⬜ | Group therapy |
|  |  | ⬜ | Partner patient |
| ⬜ | Other (please specify) | | |
|  |  | ⬜ | …....................................... |

| Indiquez tous les thèmes ou sujets que vous souhaitez aborder avec l'équipe soignante : | | | |
| --- | --- | --- | --- |
|  |  |  |  |
| ⬜ | La maladie | | |
|  |  | ⬜ | L'asthme sévère, c'est quoi ? |
|  |  | ⬜ | Les symptômes |
|  |  | ⬜ | Les idées reçues |
| ⬜ | Les traitements | | |
|  |  | ⬜ | Traitements inhalés (technique de prise, durée d'action, que faire en cas d'oublis, …) |
|  |  | ⬜ | Corticothérapie orale (cure courte/au long cours, mesures associées) |
|  |  | ⬜ | Biothérapie (quoi, qui, comment, quand, auto-injecteur) |
| ⬜ | Les crises | | |
|  |  | ⬜ | Les signes d'alerte |
|  |  | ⬜ | Que faire en cas de crise |
|  |  | ⬜ | Le contrôle de l'asthme (autosurveillance, DEP, test de contrôle de l'asthme) |
| ⬜ | La vie avec l'asthme | | |
|  |  | ⬜ | Les allergies |
|  |  | ⬜ | La pollution |
|  |  | ⬜ | Les maladies associées (maladies ORL, RGO, Obésité, apnées du sommeil) |
|  |  | ⬜ | Le sport |
|  |  | ⬜ | Les émotions |
|  |  | ⬜ | La grossesse |
|  |  | ⬜ | L'entourage (isolement social, soutien à l'aidant, …) |
|  |  | ⬜ | Les droits sociaux |
|  |  | ⬜ | Nutrition/diététique |
| ⬜ | Partage avec d'autres patients | | |
|  |  | ⬜ | Ateliers de groupe |
|  |  | ⬜ | Patient partenaire |
| ⬜ | Autre (précisez) | | |
|  |  | ⬜ | …....................................... |
