# Supplementary material for: Patient education needs in severe asthma, a pilot study
Source: BMC Pulm Med. 2024 Mar 15;24:134. doi: 10.1186/s12890-024-02960-8 (PMC10943831; doi:10.1186/s12890-024-02960-8)
Supplement: Supplementary file 4 — Supplementary Material 4 [file 12890_2024_2960_MOESM4_ESM.docx]

Supplementary Table 3. Baseline characteristics of patients who had participated in the Therapeutic Patient Education (TPE) program end those who had not.

|  | | | Previous TPE | No previous TPE | p-value |
| --- | --- | --- | --- | --- | --- |
| Number of patients | | | 21 | 32 |  |
| Age (yrs) (mean±SD) | | | 54.6 ± 12.9 | 52.8 ± 14.2 | 0.318 |
| Age at severe asthma diagnosis (yrs) (mean±SD) | | | 50.7 ± 14.5 | 49.0 ± 15.8 | 0.348 |
| Female gender (n, %) | | | 14 (66.6) | 7 (21.8) | 0.181 |
| Body mass index (kg/m²) (mean±SD) | | | 30.4 ± 4.8 | 27.8 ± 6.4 | 0.060 |
|  | ≥30 (n, %) | | 10 (47.6) | 8 (25.0) | 0.138 |
| Smoking history | | |  |  |  |
|  | Current smoker (n, %) | | 0 (0.0) | 2 (9.5) | 0.222 |
|  | Ex-smoker (n, %) | | 8 (38.0) | 16 (50.0) |  |
|  | Pack-year (mean±SD) | | 18.2 ± 16.0 | 14.6 ± 12.7 | 0.277 |
| Comorbidities | | |  |  |  |
|  | Nasal polyps (n, %) | | 11 (52.4) | 12 (37.5) | 0.397 |
|  | GERD (n, %) | | 9 (42.9) | 11 (34.3) | 0.573 |
|  | Allergic rhinitis (n, %) | | 6 (28.6) | 6 (18.8) | 0.507 |
| Occupation category | | |  |  |  |
|  | Manager and intellectual professions (n, %) | | 3 (14.3) | 5 (15.6) | 0.458 |
|  | Technical professions (n, %) | | 6 (28.6) | 14 (43.8) |  |
|  | Services (n, %) | | 9 (42.9) | 8 (25.0) |  |
|  | Unemployed (n, %) | | 3 (14.3) | 3 (9.4) |  |
|  | Students (n, %) | | 0 (0.0) | 2 (6.3) |  |
| Treatment adherence (MARS score ≥21) | | | 19 (90.4) | 29 (90.6) | 1 |
| ACT score (mean±SD) | | | 16.0 ± 5.6 | 17.8 ± 5.1 | 0.116 |
|  | <15 (n, %) | | 11 (52.4) | 6 (28.6) |  |
|  | 15-19 (n, %) | | 2 (9.5) | 12 (57.1) | 0.016 |
|  | ≥20 (n, %) | | 8 (38.1) | 14 (43.8) |  |
| Exacerbations | | |  |  |  |
|  | In the last year (n, %) | | 15 (71.4) | 20 (62.5) | 0.502 |
|  | Number per patient (median[25^th^-75^th^] | | 2 [0-4] | 1 [0-3.8] | 0.360 |
|  | ≥ 2 severe exacerbations in the last year (n,%) | | 13 (61.9) | 12 (37.5) | 0.099 |
| Blood eosinophils (10^9^/L) * (median[25^th^-75^th^] | | | 636 [300-900] | 650 [500-1245] | 0.086 |
|  | <150 (n, %) | | 12 (57.1) | 11 (34.4) | 0.178 |
|  | 150-299 (n, %) | | 2 (9.5) | 2 (6.3) |  |
|  | ≥300 (n, %) | | 7 (33.3) | 19 (59.4) |  |
| Aeroallergen sensitization (n, %) | | | 12 (57.1) | 14 (43.8) | 0.177 |
| FEV_1_ post bronchodilation, % (mean±SD) | | | 77.9 ± 17.2 | 81.6 ± 19.6 | 0.242 |
| FEV_1_/FVC (mean±SD) | | | 66.6 ± 10.1 | 69.2 ± 9.8 | 0.181 |
| Treatment at inclusion | | |  |  |  |
|  | Inhaled corticosteroid (n, %) | | 21 (100) | 32 (100) | 1 |
|  | Long-acting beta2 agonist (n, %) | | 21 (100) | 32 (100) | 1 |
|  | Long-acting muscarinic antagonist (n, %) | | 7 (33.3) | 6 (18.8) | 0.329 |
|  | Montelukast (n, %) |  | 12 (57.1) | 15 (46.9) | 0.577 |
|  | Long-term oral corticosteroid (previous or current) (n, %) | | 8 (38.1) | 8 (25.0) | 0.368 |
|  | OCS dose (mg/day) (mean±SD) | | 14.3 ± 5.1 | 11.2 ± 9.7 | 0.222 |
|  | Biologic therapy (current) (n, %) | | 18 (85.7) | 22 (68.8) | 0.204 |
|  | 1st line (n, %) | | 9 (42.9) | 11 (34.4) | 0.533 |
|  | ≥2nd line (n, %) | | 9 (42.9) | 11 (34.4) | 0.533 |
| Data are expressed as number (percentage), mean ± SD or median [25^th^-75^th^]  *: highest count before biologic treatment  GERD: Gastro-eosophageal reflux disease; MARS: Medical Adherence Report Scale; ACT: Asthma Control Test; TPE: Therapeutic patient education | | | | |  |
